# Extended Multicriteria Group Decision Making with a Novel Aggregation Operator for Emergency Material Supplier Selection

**DOI:** 10.3390/e25040702

**Published:** 2023-04-21

**Authors:** Ling Liu, Qiuyi Zhu, Dan Yang, Sen Liu

**Affiliations:** 1 School of Logistics and Management Engineering, Yunnan University of Finance and Economics, Kunming 650221, China; lingliu@ynufe.edu.cn (L.L.); 201705003916@stu.ynufe.edu.cn (Q.Z.); 2 College of Innovative Business and Accountancy, Dhurakij Pundit University, Bangkok 10210, Thailand; yangdanfiy@163.com

**Keywords:** emergency medical supplier, IFHPWA operator, entropy, COVID-19, MCGDM

## Abstract

How to ensure the normal production of industries in an uncertain emergency environment has aroused a lot of concern in society. Selecting the best emergency material suppliers using the multicriteria group decision making (MCGDM) method will ensure the normal production of industries in this environment. However, there are few studies in emergency environments that consider the impact of the decision order of decision makers (DMs) on the decision results. Therefore, in order to fill the research gap, we propose an extended MCGDM method, whose main steps include the following: Firstly, the DMs give their assessment of all alternatives. Secondly, we take the AHP method and entropy weight method to weight the criteria and the DMs. Thirdly, we take the intuitionistic fuzzy hybrid priority weight average (IFHPWA) operator we proposed to aggregate evaluation information and take the TOPSIS method to rank all the alternatives. Finally, the proposed method is applied in a case to prove its practicability and effectiveness. The proposed method considers the influence of the decision order of the DMs on the decision results, which improves the accuracy and efficiency of decision-making results.

## 1. Introduction

According to data released by the World Health Organization, as of November 2022, there have been more than 600 million confirmed cases of COVID-19 worldwide, and more than 6 million people have died of COVID-19 [1]. The outbreak of the COVID-19 pandemic was sudden, universal, and complex, and it triggered a major global public health crisis [2,3], which has led to the shortage of medical resources and even the collapse of the medical system [4,5]. Concurrently, COVID-19 has seriously affected the regular operation of the medical material supply chain [6,7,8]. With the outbreak of COVID-19, the global demand for medical supplies is expected to be approximately 100 times higher than usual according to the World Health Organization. Because the Chinese government has decreased the level of prevention and control of COVID-19, it will no longer implement isolation measures for people infected with COVID-19 and no longer delimit high- and low-risk areas. In the short term, the number of people infected with COVID-19 has increased sharply, and most have had mild and asymptomatic infections. When the demand for medical supplies rises sharply because people are infected with variants of COVID-19 in Chinese society, the corresponding material supplier cannot meet the real demand in the short term [7,9,10].

With the global pandemic of COVID-19, emergency public health events have become more frequent, seriously affecting the safety of people’s lives and property [11,12,13]. When such events occur, appropriate emergency decision-making methods are required to solve the problem to minimize its negative impact. Due to the characteristics of low efficiency, long delays, and the high cost of emergency decisions, their decision process is complex and their accuracy is difficult to control [14]. In the face of the characteristics of emergency decisions, many scholars try to empower the evaluation criteria and DMs (decision makers) to improve the accuracy of their decisions; however, the DMs often ignore the fact that their decision sequence guides their decisions’ results, sometimes even directly affecting the accuracy of the decisions’ results [15]. Many studies have investigated emergency decision making, emergency material supplier selection, emergency medical material supply, and other aspects [16,17,18]. The relevant research methods have been sorted and summarized and can be primarily divided into quantitative analysis [19,20], qualitative analysis [21], and a combination of quantitative analysis and qualitative analysis [16,18,22]. The multicriteria group decision making (MCGDM) method considers qualitative and quantitative evaluation criteria and effectively solves many real emergency decision problems.

The literature on emergency decision making using the MCGDM method usually cannot describe the characteristics of “emergency events” accurately. There are currently two types of limitations in the study of the MCGDM method for the evaluation of emergency material suppliers. The existing research on emergency decision making struggles to accurately propose and describe the evaluation criteria of its relevant emergency problems [23]. The evaluation criteria proposed by some studies do not fit the “emergency” situation. The evaluation criteria used by some studies are too general to describe emergency decision-making problems effectively and completely. The second limitation describes how the decision order of the DMs guides the decision results via their decision-making process. In the unique environment of emergency decision making, the influence of this characteristic is rapidly amplified. Many decision makers at a lower rank easily make decisions by referring to the subjective attitude of the decision makers at a higher rank for the emergency decision-making problem, which reduces the real effectiveness of group decision making [15]. Therefore, we propose an extended MCGDM method based on an intuitionistic fuzzy environment, considering the influence of decision makers’ decision order on the decision. Based on the decision-making environment of emergency material suppliers, this method proposes a relatively comprehensive and complete evaluation criteria system including 7 primary evaluation criteria and 26 secondary evaluation criteria. This includes primary evaluation criteria directly related to “emergency supply”, such as rapid response ability, supply capacity, and flexibility, which effectively meet the requirements of emergency decision making [24]. In addition, we propose a new aggregation operator, called the IFHPWA operator, which can integrate the weights of evaluation criteria and DMs and consider the influence of the decision makers’ sequences on the decision. This operator simplifies decision steps and improves decision efficiency, which is critical for emergency decisions.

There are three major contributions outlined in this paper. First, a decision-making framework that considers the decision makers’ decision sequence is proposed for optimizing the existing emergency material supplier selection research. This framework considers the decision environment of emergency decision making, simplifies the decision steps as much as possible, and ensures scientific and accurate decision making. The framework is closer to the real emergency decision-making situation and can effectively solve practical problems. Second, a new aggregation operator (IFHPWA) is developed to make the emergency decision-making process fit better with reality. The aggregation operator is one of the essential components of the MCGDM method; thus, a novel aggregation operator can effectively improve the scientific nature of decision making. The aggregation operator can describe the impact of decision makers’ decision order on decision results while aggregating the weight of the decision makers, evaluation criteria, and group decision matrix so that the decision results are more accurate. Last, a comprehensive and widely applicable emergency material supplier evaluation criteria system is presented that includes 7 primary criteria and 26 secondary criteria; thus, the evaluation results can be more consistent with a real emergency material supplier selection situation.

The rest of this paper is organized as follows. In Section 2, we introduce the relevant research methods of emergency decision making and review commonly used MCGDM methods, including weighting methods, aggregation operators, and ranking methods. Section 3 introduces the theoretical background of assessment fuzzy sets and a novel aggregation operator. In Section 4, we propose a novel evaluation criteria system for emergency material supplier selection. In Section 5, we discuss the methods and implementation steps proposed in this paper. In Section 6, we apply the method mentioned in Section 5 to practical cases and verify the validity of this method. In Section 7, we discuss the research significance and application value of the proposed method in this paper. Finally, the conclusion of the study is provided in Section 8.

## 2. Literature Review

### 2.1. Decision Making in Emergency Material Suppliers Selection

With the global COVID-19 pandemic, public health emergencies around the world occur frequently, and the demand for emergency supplies has increased rapidly, particularly for medical protective equipment [25]. For example, Shanghai, Wuhan, and Chengdu, which are three large, modern cities in China, exposed the lack of emergency supply management capacity due to the massive spread of the COVID-19 epidemic [26]. The supply situation of emergency supplies in other regions of China is more severe [27,28]. Therefore, in the foreseeable environment of the rapid spread of the COVID-19 epidemic, it is difficult to meet the need for various emergency supplies in time.

To respond to the demand for emergency supplies in time, many studies have investigated the emergency system mechanism, the supply side of emergency material, the locations of emergency facilities, and the reserve of emergency supplies from multiple perspectives and fields [17,20,29,30,31,32,33] and proposed evaluation criteria for emergency management systems, optimizing the supply chain of emergency supplies, selecting appropriate emergency supply suppliers, and other methods to meet the demand for the timely supply of emergency material [6,14,15,34,35]. Research on emergency material supplier selection can be applied to practical problems with a shorter response time, providing quick results at lower decision-making costs; this process has gradually become one of the mainstream trends in emergency response research [17].

In existing research on emergency supplier selection, scholars have used various decision-making tools and methods. Some scholars have used many quantifiable mathematical models to conduct in-depth research on foreseeable supply interruptions, uncertain supply times, limited supply market resources, unreliable suppliers, and other risks in emergency supplies. For example, the MINLP model has been proposed for joint supplier selection under supply interruption risk [36]. In addition, suppliers’ probability of emergency supply disruption has been discussed, and a real-time updated emergency decision-making model has been proposed based on the Stackelberg game and Bayesian inference [37]. Moreover, some scholars analyze emergency decision making through purely qualitative methods. Examples include the dissemination mechanism of health-related misinformation about COVID-19 by drawing on social support theory and text mining [38], field research on COVID-19 and its related subjective distress in society, and the long-term impact of the COVID-19 epidemic on the mental state of the social population [39]. Compared to other methods, a purely quantitative method or purely qualitative method may have lower persuasive power [40,41]. Thus, a combination of quantitative and qualitative analysis methods is more scientific and effective [16,18]. Some of the methods applied in emergency decisions are listed in Table 1.

As shown in Table 1, the MCGDM method has become a mainstream method when studying emergency decision making. However, few scholars have studied the selection of emergency medical material suppliers using MCGDM methods [17].

By reviewing the relevant research on emergency decision making and emergency material supplier selection, we found that such research has high theoretical importance and practical value. In addition, the primary methods of such research can be summarized in three types: qualitative analysis, quantitative analysis, and a combination of qualitative and quantitative analysis. The combination of qualitative and quantitative analysis is more scientific and convincing. The MCGDM method simultaneously considers both qualitative and quantitative analysis and has become one of the mainstream methods in research on emergency decision making. However, few studies have investigated the selection of emergency medical material suppliers; thus, this topic still requires further exploration and discussion.

### 2.2. MCGDM Method

**Figure 1:**
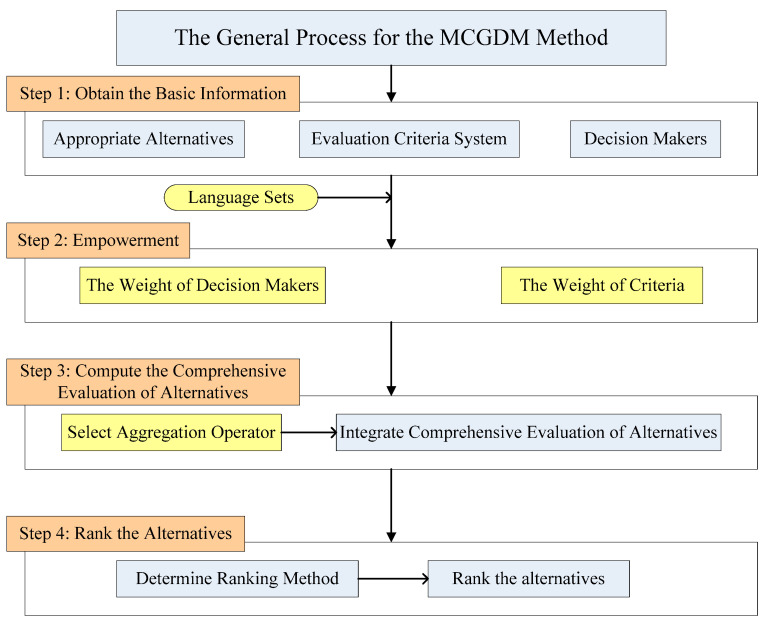
General process for solving the MCGDM method.

The MCGDM method combines qualitative and quantitative analysis and is one of the commonly used methods in research on emergency decision making [14,18,44]. The MCGDM method decision-making process primarily consists of four steps (refer to Figure 1): (1) confirm the decision-makers, clarify the evaluation criteria system, and provide appropriate alternatives; (2) determine the weights of the decision makers and the weights of the criteria; (3) select the aggregation operator to integrate a comprehensive evaluation of the alternatives; and (4) rank the alternatives using the determined ranking method.

In the MCGDM method, each sub-method has its own advantages and disadvantages. Some research has explored the improvement or combination of various sub-methods to complement the advantages and disadvantages of the methods and make the decision results more scientific and accurate. There are three primary improvement directions: language sets [45,46], weighting assignment [47], and aggregation operators [43,48]. The following is a brief summary of the current scholars’ discussion and exploration of the three primary improvement directions of the MCGDM method in research on emergency decision making.

Different language sets have different degrees of information distortion and loss in the description of the real situation; thus, some studies have analyzed which language set can better fit a real situation [49]. Since Zadeh first proposed the concept of fuzzy sets in 1965 [50], it has been proven that compared with clear sets, fuzzy sets can better describe decision-making problems and evaluation objects in the face of uncertainty in the decision-making environment and the complexity of individual decision makers [51]. To adapt to the complexity and variability of the decision-making process, researchers have developed different types of fuzzy sets, such as intuitionistic fuzzy sets [52], triangular fuzzy sets [43], trapezoid fuzzy sets [53], probabilistic hesitant fuzzy sets [54], interval type-2 fuzzy sets [17], etc. Through practical application and theoretical verification, intuitionistic fuzzy sets have been shown to have high stability and wide applicability [55]. In emergency decision making with high requirements for time cost and economic cost, it is essential to accurately describe the DMs’ attitude toward the decision object [56].

Evaluation of the importance degree of decision makers and criteria is a key part of research on the MCGDM method. Weighting methods can be divided into subjective weights and objective weights [57]. Subjective weighting methods apply mathematical methods to determine weights based on the preferences of decision makers. There are several representative objective weighting methods: the decision-making trial and evaluation laboratory (DEMATEL) method, the analytic hierarchy process (AHP) method [57], the best and worst method (BWM), and the analytic network process (ANP) method [58,59,60,61]. The objective weighting method determines weights by automatically solving mathematical models without considering the preferences of decision makers. There are several representative subjective weighting methods: the vlse kriterijumski optimizacioni racun (VIKOR) method, the technique for order preference by similarity to an ideal solution (TOPSIS) method, the deviation maximization method, and the entropy weight method [62,63,64,65,66]. 

In the final grading and ranking stage of alternatives, the primary methods for aggregating decision information in an intuitionistic fuzzy environment include the intuitionistic fuzzy weighted geometric (IFWG) operator, IF ordered weighted geometric (IFOWG) operator, IF weighted averaging (IFWA) operator, and IF ordered weighted average (IFOWA) operator [67,68,69]. When aggregating various interval numbers, fuzzy language sets, and other information, fuzzy information is always quantified somehow and then becomes deterministic data, while this process is accompanied by a certain degree of information attenuation [70,71]. Therefore, how to make the aggregated information as complete as possible and add additional information to fill the information loss in the decision-making process has become a novel research direction in the field of aggregation operators in MCGDM [72,73,74].

Through a summary and analysis of the existing relevant literature, the intuitionistic fuzzy language set is shown to be more suitable for the needs of a real emergency decision-making environment. At the stage of gathering decision information, there is little research on reducing information loss after the quantification of decision information and adding additional information to fill in gaps in decision information at this stage.

## 3. Theoretical Background

### 3.1. Intuitionistic Fuzzy Set

Due to the complexity of the evaluation and the ambiguity of the existing information, decision makers often show some hesitation in decision making. Atanassov [52] proposed the concept of an intuitive fuzzy set, which accounts for the information hesitancy degree of decision makers in the decision-making process. This section focuses on some definitions and algorithms for intuitionistic fuzzy sets.

We let X be a non-empty set and A be a subset of X:

$$A=\left\{\langle x,\mu_{A}(x),\nu_{A}(x)\rangle |x\in X\right\}$$

This set is an intuitionistic fuzzy set, where $\mu_{A}(x)$ and $\nu_{A}(x)$ are the membership function and non-membership function of the intuitionistic fuzzy set A:

$$\begin{array}{l} \mu_{A}:X\to [0,1],x\in X\to \mu_{A}(x)\in [0,1]\\ \nu_{A}:X\to [0,1],x\in X\to \nu_{A}(x)\in [0,1] \end{array}$$

with the condition of

$$0\leq \mu_{A}(x)+\nu_{A}(x)\leq 1$$

If $\pi_{A}(x)=1-\mu_{A}(x)-\nu_{A}(x)$, then $\pi_{A}(x)$ is used to measure the hesitancy degree in intuitionistic fuzzy sets.

The intuitionistic fuzzy numbers that are composed of membership degree, non-membership degree, and hesitation degree can be expressed as r=(μ,ν,π). For any two IFSs, $r_{1}=(\mu_{1},\nu_{1},\pi_{1})$ and $r_{2}=(\mu_{2},\nu_{2},\pi_{2})$; the positive real number λ, λ≥0; and the operational laws of $r_{1}$, $r_{2}$, and which can be defined as follows:

(1) $$r_{1}⊕r_{2}=(\mu_{1}+\mu_{2}-\mu_{1}\mu_{2},\nu_{1}\nu_{2},(1-\mu_{1})(1-\mu_{2})-\nu_{1}\nu_{2})$$

(2) $$r_{1}⊗r=(\mu_{1}\mu_{2},\nu_{1}+\nu_{2}-\nu_{1}\nu_{2},(1-\nu_{1})(1-\nu_{2})-\mu_{1}\mu_{2})$$

(3) $$\lambda r_{1}=(1-{\left(1-\mu_{1}\right)}^{\lambda },\nu_{1}{}^{\lambda },{\left(1-\mu_{1}\right)}^{\lambda }-\nu_{1}^{\lambda }),\lambda >0$$

(4) $$r_{1}{}^{\lambda }=(\mu_{1}{}^{\lambda },1-{\left(1-\nu_{1}\right)}^{\lambda },{\left(1-\nu_{1}\right)}^{\lambda }-\mu_{1}{}^{\lambda }),\lambda >0$$

(5) $$r_{1}\cap r_{2}=\left\{\min\left\{\mu_{1},\mu_{2}\right\},\max\left\{\nu_{1},\nu_{2}\right\},1-\min\left\{\mu_{1},\mu_{2}\right\}-\max\left\{\nu_{1},\nu_{2}\right\}\right\}$$

(6) $$r_{1}\cup r_{2}=\left\{\max\left\{\mu_{1},\mu_{2}\right\},\min\left\{\nu_{1},\nu_{2}\right\},1-\max\left\{\mu_{1},\mu_{2}\right\}-\min\left\{\nu_{1},\nu_{2}\right\}\right\}$$

The Euclidean distance is as follows:

(7) $$d_{E}(r_{1},r_{2})=\sqrt{\frac{1}{2}\sum_{j=1}^{n}\left[{\left(\mu_{r_{1}}(x_{j})-\mu_{r_{2}}(x_{j})\right)}^{2}+{\left(v_{r_{1}}(x_{j})-v_{r_{2}}(x_{j})\right)}^{2}+{\left(\pi_{r_{1}}(x_{j})-\pi_{r_{2}}(x_{j})\right)}^{2}\right]}$$

The normalized Euclidean distance is obtained by the following:

(8) $$d_{nE}(r_{1},r_{2})=\sqrt{\frac{1}{2n}\sum_{j=1}^{n}\left[{\left(\mu_{r_{1}}(x_{j})-\mu_{r_{2}}(x_{j})\right)}^{2}+{\left(v_{r_{1}}(x_{j})-v_{r_{2}}(x_{j})\right)}^{2}+{\left(\pi_{r_{1}}(x_{j})-\pi_{r_{2}}(x_{j})\right)}^{2}\right]}$$

where n represents the number of evaluation criteria or objects.

### 3.2. Entropy

In information theory, entropy is a measure of uncertainty. Shannon proposed the concept of information entropy, using the characteristics of entropy to calculate the weight of each index or the weight of each decision maker according to the degree of variation in each index or the individual judgment matrix of each decision maker to provide a basis for the comprehensive evaluation of multiple criteria.

Bustince and Burillo [64] extended the fuzzy nonprobability entropy measure formula to the intuitionistic fuzzy set, proposing that when the entropy measure E(r) satisfies the following conditions, it can be called intuitionistic fuzzy entropy:

1. E(r)=0 if and only if r∈FSs(X), where FSs(X) represents the set of all fuzzy sets on X;
2. E(r)=1 if and only if $\mu_{A}(x)=\nu_{A}(x)=0$ for all x∈X;
3. If the $r_{1}$ fuzzy degree is lower than $r_{2}$, then $E(r_{1})\leq E(r_{2})$;
4. $E(r)=E(r_{c})$ for all r∈IFSs(X), where IFSs(X) represents the set of all the intuitionistic fuzzy sets on X.

### 3.3. Improved IFHPWA Aggregation Operator

The intuitionistic fuzzy priority weight averaging (IFPWA) operator and the intuitionistic fuzzy priority weight geometry (IFPWG) operator are weighted only based on the priority level between the criteria, without considering the importance of intuitionistic fuzzy sets. The intuitionistic fuzzy weighted arithmetic average (IFWAA) operator and intuitionistic fuzzy weighted geometry average (IFWGA) operator are weighted according to the importance of intuitionistic fuzzy sets, ignoring the priority level between the criteria. The above aggregation operators have marked limitations in use [67,75]. Therefore, we propose the intuitionistic fuzzy hybrid priority weight average (IFHPWA) operator, which combines the advantages of the IFPWA operator and the IFWAA operator considering the importance of the intuitionistic fuzzy sets and the priority of criteria.

Let $r_{1}=(\mu_{1},\nu_{1},\pi_{1})$ be an intuitionistic fuzzy number, and use the score function to compute the score value of the intuitionistic fuzzy number r:

(9) $$S(r_{1})=\frac{1+\mu_{1}-\nu_{1}}{2}$$

For S(a)∈[0,1], let $r_{1}=(\mu_{1},\nu_{1},\pi_{1})$ and $r_{2}=(\mu_{2},\nu_{2},\pi_{2})$ be two intuitionistic fuzzy numbers, which can be defined as follows:

1. $S(r_{1})<S(r_{2})\to r_{1}<r_{2}$.
2. $S(r_{1})\geq S(r_{2})\to r_{1}\geq r_{2}$.

There are three steps in the improved IFHPWA aggregation operator:

1. A normalized individual decision matrix is weighted.
2. The priority level of decision makers is considered.
3. The comprehensive evaluation decision matrix is obtained.

## 4. Evaluation Criteria of Emergency Medical Suppliers

According to the characteristics of emergency goods, combined with field investigation and consideration of a large number of relevant studies, we propose the evaluation criteria system of emergency material suppliers, as shown in Table 2, which includes 7 primary criteria (enterprise credit ($C_{1}$), rapid response ability ($C_{2}$), quality ($C_{3}$), supply capacity ($C_{4}$), flexibility ($C_{5}$), price ($C_{6}$), and enterprise environment ($C_{7}$)) and 26 secondary criteria.

There are eight secondary criteria under enterprise credit ($C_{1}$), in addition to the criteria commonly used to evaluate an enterprise’s internal and external operational status: financial status, development prospects, industry status and reputation, equipment level, cooperation attitude, and employee quality [76,77]. In terms of the selection of emergency material suppliers, risk management ability and informatization degree are two very important standards, especially when a firm operates its business within an uncertain environment [78]. For emergency material suppliers, excellent risk management ability can manage the uncertainty, complicacy, and fuzziness of the information environment in public health emergencies more effectively [18]. The purpose of evaluating the informatization degree of emergency material suppliers is to determine whether they have the capacity to quickly interact with enterprises in the supply chain [79] to reduce communication costs and improve communication efficiency in the supply chain.

There are three secondary criteria under the rapid response ability ($C_{2}$), including information exchange capacity, emergency distribution capacity, and order processing ability, which can comprehensively and meticulously describe the material distribution and allocation capabilities of emergency material suppliers [80,81]. Information in information exchange capacity includes described useable data, inferences from data, or data descriptions [82]. This information is processed at multiple degrees before being used for decisions, so the firm may lack access to some of the information that is vital for its strategic decision making [83]. Therefore, information exchange capacity can form the basis for developing a competitive advantage. Emergency distribution capacity and order processing ability reflect the operational resilience of enterprises in uncertain environments [84].

There are four secondary criteria under quality ($C_{3}$): return processing capacity, qualified rate, quality certification, and total quality management ability. Return processing capacity mainly tests the reverse logistics transportation ability of a firm, which is crucial to protecting the interests of the purchaser [85]. Qualified rate, quality certification, and total quality management ability are three criteria relationships that influence each other. Quality certification is the basis for product qualification rate. Excellent total quality management ability ensures that the quality certification process is controllable and efficient and that the product qualification rate can be maintained at a high level [86,87].

Supply capacity ($C_{4}$) is measured by three criteria: order completion rate, packing sound rate, and delivery punctuality. Supply capacity originally also included rapid response ability, but due to the purchase of emergency goods, we need to ensure the evaluation of emergency material suppliers is more accurate and practical; therefore, we made rapid response capability a primary criterion. In the emergency environment, the supply capacity of a firm is not only production capacity in a short period of time [88], but also the arrival quality and timely arrival of products [89]. Therefore, we use these three secondary criteria to describe supply capacity in emergency and uncertain environments.

Flexibility ($C_{5}$) includes the three secondary criteria of batch flexibility, time flexibility, and category flexibility. Flexibility is a concept derived from the need to reduce adverse effects on the supply chain [90], which is identified as the capability of a supply chain to be resilient and to react and change in order to meet changes in market demand [91]. Demanders and suppliers are important components of the supply chain, so the flexibility of suppliers in terms of batches [10], time [92], and categories has a significant impact on the demand side [93].

Price ($C_{6}$) includes the two secondary criteria of price stability and price advantage. The timeliness of emergency goods and the demand resilience of emergency material is strong [29], so its price changes frequently and is generally at a high level. In proposing the evaluation criteria of price stability and price advantage, our purpose is to control procurement costs [94] and to judge the stability [95] and cost performance of supply channels and the cooperation intentions of suppliers.

The enterprise environment ($C_{7}$) has three secondary criteria: the natural environment, the economic environment, and the political environment. From a macroscopic aspect, the three criteria can comprehensively evaluate the environment of the enterprise [92], of which the latter two have a strong impact on the enterprise in the context of emergency material procurement [96,97]; if these environmental conditions are insufficient, they will even directly affect the implementation of the emergency procurement contract [98]. Therefore, this topic must be carefully studied and judged to prevent huge risks from affecting the production and operation of enterprises (see Table 2).

## 5. Proposed Method

**Figure 2:**
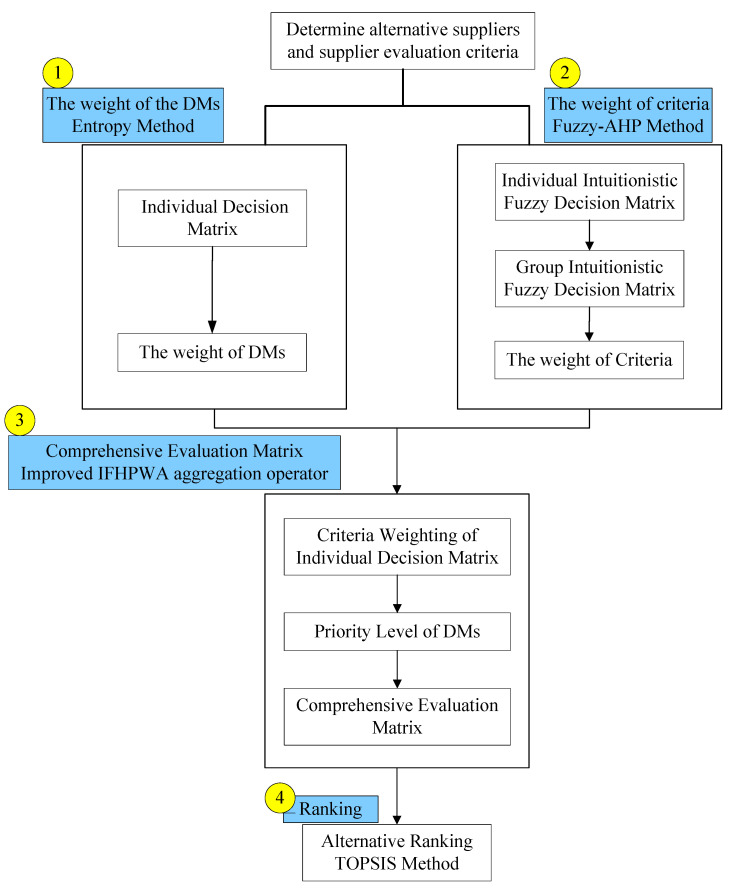
Conceptual framework of the research methodology.

We aimed to solve classical and vital emergency material supply problems with new combinational methods. An emergency material supplier evaluation method based on the intuitionistic fuzzy environment is proposed, with weighted evaluation criteria used by the improved AHP method, weighted DMs determined by the entropy method, and the IFPHWA aggregation operator used to aggregate the evaluation of the DMs. Finally, we rank the comprehensive evaluation of suppliers using the TOPSIS method. The specific process is shown in Figure 2. The symbols used in the method in this article are shown in Table 3.

1. An individual decision matrix is constructed and aggregated into a group decision matrix to determine the weight of the DMs.

 (1) An individual decision matrix is built.

Every decision maker appraises each alternative according to the criteria, thereby forming an individual decision matrix $R_{k}$:

$$R_{k}={\left(R_{ij}^{k}\right)}_{m\times n}=\left(\begin{array}{ccc} r_{11}^{k} & \ldots & r_{1n}^{k}\\ \vdots & \ddots & \vdots \\ r_{m1}^{k} & \cdots & r_{mm}^{k} \end{array}\right)=\left(\begin{array}{ccc} \left(\mu_{11}^{k},\nu_{11}^{k},\pi_{11}^{k}\right) & \ldots & \left(\mu_{1n}^{k},\nu_{1n}^{k},\pi_{1n}^{k}\right)\\ \vdots & \ddots & \vdots \\ \left(\mu_{m1}^{k},\nu_{m1}^{k},\pi_{m1}^{k}\right) & \cdots & \left(\mu_{mn}^{k},\nu_{mn}^{k},\pi_{mn}^{k}\right) \end{array}\right)$$

 (2) The weight of the DMs is determined using the entropy method:

(10) $$E^{IFS}(R_{k})=\frac{1}{mn\ln2}\sum_{i=1}^{m}\sum_{j=1}^{n}\left[\mu_{ij}\ln\mu_{ij}+\nu_{ij}\ln\nu_{ij}-(1-\pi_{ij})\ln(1-\pi_{ij})-\pi_{ij}\ln2\right]$$

The degree of divergence $d_{R^{k}}$ is then calculated. When $\mu_{ij}=0,\nu_{ij}=0,\pi_{ij}=1$, $\mu_{ij}\ln\mu_{ij}=0,\nu_{ij}\ln\nu_{ij}=0,(1-\pi_{ij})\ln(1-\pi_{ij})=0$, where j=1,2,⋯,n; i=1,2,⋯,m:

(11) $$d_{R_{k}}=1-E^{IFS}(R_{k})$$

Through calculation of the degree of divergence, the weight of the DMs is obtained:

(12) $$w_{d}^{k}=\frac{d_{R_{k}}}{\sum_{k=1}^{p}d_{R_{k}}}$$

By calculating Equations (10)–(12) above, we can obtain the weight set of the DMs: $w_{d}=\left\{w_{d}^{1},w_{d}^{2},\cdots ,w_{d}^{p},\right\}$, where $w_{d}^{k}>0,k=1,2,\cdots ,p$ and $\sum_{k=1}^{p}w_{d}^{k}=1$.

2. An intuitionistic fuzzy decision matrix is established to determine the weight of the criteria.

 (1) An individual intuitionistic fuzzy decision matrix is established:

$$R^{k}={\left(r_{jh}^{k}\right)}_{n\times n}=\left(\begin{array}{ccc} r_{11}^{k} & \ldots & r_{1n}^{k}\\ \vdots & \ddots & \vdots \\ r_{n1}^{k} & \cdots & r_{nn}^{k} \end{array}\right)=\left(\begin{array}{ccc} \left(\mu_{11}^{k},\nu_{11}^{k},\pi_{11}^{k}\right) & \ldots & \left(\mu_{1n}^{k},\nu_{1n}^{k},\pi_{1n}^{k}\right)\\ \vdots & \ddots & \vdots \\ \left(\mu_{n1}^{k},\nu_{n1}^{k},\pi_{n1}^{k}\right) & \cdots & \left(\mu_{nn}^{k},\nu_{nn}^{k},\pi_{nn}^{k}\right) \end{array}\right)$$

where the individual intuitionistic fuzzy decision matrix expresses that a decision maker thought the importance of indicator s was greater than that of indicator j.

 (2) The consistency is checked.

We let the intuitionistic fuzzy decision matrix $R^{k}={\left(r_{jh}^{k}\right)}_{n\times n}$, where $r_{jh}^{k}=(\mu_{jh}^{k},\nu_{jh}^{k},\pi_{jh}^{k})$. When h>j+1, we let ${\bar{r}}_{jh}=({\bar{\nu }}_{jh},{\bar{\mu }}_{jh},{\bar{\pi }}_{jh})$, and then obtain the following:

(13) $${\bar{\mu }}_{jh}=\frac{\sqrt[{h-j-1}]{\prod_{t=j+1}^{h-1}\mu_{jt}\mu_{th}}}{\sqrt[{h-j-1}]{\prod_{t=j+1}^{h-1}\mu_{jt}\mu_{th}}+\sqrt[{h-j-1}]{\prod_{t=j+1}^{h-1}\left(1-\mu_{jt})(1-\mu_{th}\right)}}$$

(14) $${\bar{v}}_{jh}=\frac{\sqrt[{h-j-1}]{\prod_{t=j+1}^{h-1}\nu_{jt}\nu_{th}}}{\sqrt[{h-j-1}]{\prod_{t=j+1}^{h-1}\nu_{jt}\nu_{th}}+\sqrt[{h-j-1}]{\prod_{t=j+1}^{h-1}\left(1-\nu_{jt})(1-\nu_{th}\right)}}$$

When h=j+1 or h=j, then ${\bar{r}}_{jh}=r_{jh}^{k}$; when h<j, then ${\bar{r}}_{jh}=r_{jh}^{k}$; when h<j, then ${\bar{r}}_{jh}=({\bar{\nu }}_{jh},{\bar{\mu }}_{jh},{\bar{\pi }}_{jh})$.

If $d(R,\bar{R})<\xi$, then the consistency of the intuitionistic fuzzy decision matrix $R^{k}$ is acceptable, where ξ is the threshold of the consistency check, generally considered to be ξ=0.1; d measures the distance between $R^{k}$ and $\bar{R}$. Thus,

(15) $$d(R^{k},\bar{R})=\frac{1}{2(n-1)(n-2)}\sum_{j=1}^{n}\sum_{h=1}^{n}\left(\left|{\bar{\mu }}_{jh}-\mu_{jh}^{k}\right|+\left|{\bar{v}}_{jh}-\nu_{jh}^{k}\right|+\left|{\bar{\pi }}_{jh}-\pi_{jh}^{k}\right|\right)$$

Conversely, when $d(R^{k},\bar{R})\geq \xi$, which indicates that the consistency of the intuitionistic fuzzy decision matrix $R^{k}$ is unacceptable, it should be appropriately modified to ensure the consistency of the judgment.

 (3) The group intuitionistic fuzzy decision matrix R is built:

(16) $$R=\frac{\sum_{k=1}^{p}R^{k}}{p}$$

 (4) The weight of the criteria is then determined.

According to the group intuitionistic fuzzy decision matrix R, the weight vector $w=\left[w_{1},w_{2},\cdots ,w_{n}\right]$ of each indicator at the same level can be determined using Equation (17):

(17) $$w_{c}=\left[\frac{\sum_{h=1}^{n}\mu_{jh}}{\sum_{j=1}^{n}\sum_{h=1}^{n}\left(1-\nu_{jh}\right)},1-\frac{\sum_{h=1}^{n}\left(1-\nu_{jh}\right)}{\sum_{j=1}^{n}\sum_{h=1}^{n}\mu_{jh}}\right]$$

The combined weights can be calculated as follows:

(18) $$W_{jh}=\overset{n}{\underset{j=1}{⊕}}(w_{c_{jh}}⊗w_{c_{j}}),j=1,2,\cdots ,n$$

Therefore, the combined weight set of the criteria is obtained: $w_{jh}=\left\{w_{11},w_{12},\cdots ,w_{nn}\right\}$.

3. Using IFHPWA integrates the comprehensive evaluation for alternatives.

The intuitionistic fuzzy hybrid priority weighted average (IFHPWA) operator is used to assemble the decisionmaker weights and criteria weights and considers the decision makers’ priority level to obtain the comprehensive decision matrix D.

 (1) The individual decision matrix is weighted.

The normalized individual decision-making matrix is weighted by Equation (19), including decision-maker weights $w_{d}^{k}$ and criteria weight $W_{jh}$. $R_{d}^{k}$ represents the weighted individual decision matrix:

(19) $$R_{d}^{k}=w_{d}^{k}⊗(n⊗W_{jh}⊗R_{k})$$

 (2) The decision makers’ priorities are considered.

The decision makers’ priority is taken into the overall evaluation by calculating Equation (20):

(20) $$T_{ij}^{\left(1\right)}=1,T_{ij}^{q}=\prod_{k=1}^{q-1}S(R_{d}^{k}),q\geq 2$$

 (3) A comprehensive evaluation decision matrix is constructed:

(21) $$D=IFHPWA(r_{ij}^{1},r_{ij}^{2},\cdots ,r_{ij}^{p})=\left[1-\prod_{k=1}^{p}{\left(1-\mu_{ij}^{k}\right)}^{\frac{T_{ij}^{k}}{\sum_{k=1}^{p}T_{ij}^{k}}},\prod_{k=1}^{p}{\left(\nu_{ji}^{k}\right)}^{\frac{T_{ij}^{k}}{\sum_{k=1}^{p}T_{ij}^{k}}},\prod_{k=1}^{p}{\left(1-\mu_{ij}^{k}\right)}^{\frac{T_{ij}^{k}}{\sum_{k=1}^{p}T_{ij}^{k}}}-\prod_{k=1}^{p}{\left(\nu_{ji}^{k}\right)}^{\frac{T_{ij}^{k}}{\sum_{k=1}^{p}T_{ij}^{k}}},\right]$$

4. The alternatives are ranked using the TOPSIS method.

The intuitionistic fuzzy positive and negative ideal solutions are then calculated.

The intuitionistic fuzziness positive ideal solution is $Z_{j}^{+}$, where $r_{j}^{+}=\left(\mu_{j}^{+},v_{j}^{+},\pi_{j}^{+}\right)$ represents the optimal value of evaluation criterion $C_{j}$. Among them, $\mu_{j}^{+}$ and $\nu_{j}^{+}$ are the membership and non-membership functions for evaluation criterion $C_{j}$, and $\pi_{j}^{+}$ represents hesitancy degree for evaluation criterion $C_{j}$.

(22) $$Z_{j}^{+}=(r_{1}^{+},r_{2}^{+},\cdots ,r_{n}^{+}),r_{j}^{+}=\left(\mu_{j}^{+},v_{j}^{+},\pi_{j}^{+}\right),j=1,2,\cdots ,n$$

The intuitionistic fuzzy negative ideal solution is $Z_{j}^{-}$, where $r_{j}^{-}=\left(\mu_{j}^{-},v_{j}^{-},\pi_{j}^{-}\right)$ represents the worst value of evaluation criterion $C_{j}$. Among them, $\mu_{j}^{-}$ and $\nu_{j}^{-}$ are the membership and non-membership functions for evaluation criterion $C_{j}$, and $\pi_{j}^{-}$ represents hesitancy degree for evaluation criterion $C_{j}$.

(23) $$Z_{j}^{-}=(r_{1}^{-},r_{2}^{-},\cdots ,r_{n}^{-}),r_{j}^{-}=\left(\mu_{j}^{-},v_{j}^{-},\pi_{j}^{-}\right),j=1,2,\cdots ,n$$

The distance between each alternative and the positive and negative ideal solutions is calculated separately:

(24) $$d_{i}^{+}(r_{ij},Z_{j}^{+})=\sqrt{\frac{1}{2}\times \sum_{j=1}^{n}\left[{\left(\mu_{ij}-\mu_{j}^{+}\right)}^{2}+{\left(v_{ij}-v_{j}^{+}\right)}^{2}+{\left(\pi_{ij}-\pi_{j}^{+}\right)}^{2}\right]}$$

(25) $$d_{i}^{-}(r_{ij},Z_{j}^{-})=\sqrt{\frac{1}{2}\times \sum_{j=1}^{n}\left[{\left(\mu_{{}_{ij}}-\mu_{j}^{-}\right)}^{2}+{\left(v_{{}_{ij}}-v_{j}^{-}\right)}^{2}+{\left(\pi_{{}_{ij}}-\pi_{j}^{-}\right)}^{2}\right]}$$

Finally, the relative closeness coefficient of the intuitionistic fuzzy ideal solution is calculated by the following formula and each alternative is ranked:

(26) $$CC_{i}=\frac{d_{i}^{-}}{d_{i}^{+}+d_{i}^{-}},i=1,2,\cdots ,m$$

## 6. Illustrative Example

### 6.1. Case Description

According to an announcement issued by the National Health Commission of China on 8 January 2023, novel coronavirus infections will no longer be included in quarantine infectious disease management under the Frontier Health Quarantine Law of the P.R.C. With the downregulation of the management standards for COVID-19 infections, the number of people infected with COVID-19 has increased rapidly in various parts of China. Most people infected with COVID-19 have symptoms such as a fever, a dry cough, shortness of breath, muscle pain, fatigue, a sore throat, and anosmia [99].

Enterprise A is a Guangdong pharmaceutical enterprise that produces antiviral drugs. Due to the change in the anti-epidemic policy of China, the number of people infected with COVID-19 has increased rapidly, but most are mildly or asymptomatically infected. Therefore, the demand for antiviral drugs has increased rapidly. Enterprise A decided to immediately organize its employees to produce the large number of antiviral drugs needed to meet demand. To ensure epidemic prevention safety in the production process, Enterprise A must purchase a batch of emergency anti-epidemic goods. After negotiation between the business owners and experts, the category and quantity of emergency anti-epidemic goods were determined (see Table 4).

After a comprehensive analysis by business owners and experts, three suppliers that can provide the above emergency anti-epidemic goods were finally identified: Z_1_, Z_2_, and Z_3_. The basic information of the three enterprises is briefly introduced as follows.

Z_1_ is a foreign trade export enterprise located in Japan that has been engaged in the business-to-business agent procurement business for many years. Z_1_ has been cooperating long-term with many enterprises in Japan and Southeast Asia and can quickly provide the anti-epidemic goods required by Enterprise A. However, Z_1_ cannot guarantee that all purchased goods can be delivered from Japan and cannot ensure a uniform arrival time. Z_1_ only promises to gather all purchased goods within a week and arrange the shipment as soon as possible, ensuring that all purchased goods are delivered to the receiving address of Enterprise A by air or road transportation within 25 days after they are purchased, except for force majeure. Concurrently, because it is an agency procurement business, Z_1_ guarantees that the manufacturer of the purchased goods meets the qualification requirements of Enterprise A; however, it is not acceptable to return goods based on quality inspection failure.

Z_2_ is a subsidiary of a large state-owned enterprise in China, which can complete the independent production of 80% of the goods in the purchase list of Enterprise A, and the remaining 20% are produced by another subsidiary of the state-owned enterprise, finally completing the unified delivery. The two subsidiaries are located in the same production park. Because the domestic epidemic is at the outbreak stage at this time, there are many production orders. Z_2_ said that it would try to complete the production of Enterprise A’s orders within two weeks and would concurrently cooperate with another subsidiary to ensure that the total order would be completed within 20 days. Z_2_ also indicated that it would arrange delivery to Enterprise A as soon as possible, except for force majeure. The goods will be delivered to the receiving address of Enterprise A within 10 days. Z_2_ accepts the sampling inspection of Enterprise A and allows the return of substandard products, while the return process is cumbersome.

Z_3_ is a private enterprise in China with a good reputation in the industry. The information system construction in the company has become increasingly complete, and its informatization degree has reached the domestic advanced level due to its strong information processing ability. Z_3_ said that it could deliver 60% of the goods in Enterprise A’s purchase list within 10 days and complete the delivery of the remaining purchased items within the following two weeks. Influenced by the epidemic of infections in the region where Z_3_ was located during the earlier stage, the cash flow of the enterprise was relatively tight; thus, Z_3_ proposed that after delivering the first batch of purchased items, in addition to the initial deposit, Enterprise A should pay 80% of the balance before subsequent production by Z_3_. The return service process for products with substandard quality is simpler than it is for Z_2_.

### 6.2. Case Calculation

According to the descending order of the decision makers’ priorities, the three decision makers are $\alpha_{1}$, $\alpha_{2}$, and $\alpha_{3}$. $\alpha_{1}$ is a purchasing director of Enterprise A who is responsible for the procurement of emergency goods. $\alpha_{2}$ is an external emergency management expert of Enterprise A. $\alpha_{3}$ is a senior technician who understands the internal production of Enterprise A. According to Table 5, $\alpha_{1}$, $\alpha_{2}$, and $\alpha_{3}$ appraise Z_1_, Z_2_, and Z_3_ based on the above 26 criteria. According to relevant studies [63,100,101,102,103,104] and the context of the emergency material supplier selection problem, we use 10 language variables (ranging from Excellent to Very, Very Bad) and make decision makers choose the language variable that best fits the corresponding alternative for evaluation. All calculations in this paper are based on Matlab 2018a software.

1. An individual decision matrix is constructed and aggregated into a group decision matrix to determine the weight of the DMs.

 (1) An individual decision matrix was built based on individual evaluation results, as shown in Table 6.

After processing the data in Table 5, the following normalized individual decision matrix can be obtained:

$$\begin{array}{l} R_{1}=[A_{1}B_{1}C_{1}D_{1}E_{1}]\\ A_{1}=\left[\begin{array}{l} \left(0.80,0.10,0.10)(0.80,0.10,0.10)(0.70,0.20,0.10)(0.80,0.10,0.10)(0.70,0.20,0.10)(0.50,0.40,0.10\right)\\ \left(0.80,0.10,0.10)(0.80,0.10,0.10)(0.80,0.10,0.10)(0.90,0.10,0.00)(0.50,0.40,0.10)(0.80,0.10,0.10\right)\\ \left(0.25,0.60,0.15)(0.50,0.40,0.10)(0.90,0.10,0.00)(0.80,0.10,0.10)(0.70,0.20,0.10)(0.80,0.10,0.10\right) \end{array}\right]\\ B_{1}=\left[\begin{array}{l} \left(0.80,0.10,0.10)(0.90,0.10,0.00)(0.80,0.10,0.10)(0.90,0.10,0.00)(0.70,0.20,0.10)(0.10,0.90,0.00\right)\\ \left(0.80,0.10,0.10)(0.70,0.20,0.10)(0.90,0.10,0.00)(0.70,0.20,0.10)(0.50,0.40,0.10)(0.80,0.10,0.10\right)\\ \left(0.70,0.20,0.10)(0.80,0.10,0.10)(0.80,0.10,0.10)(0.80,0.10,0.10)(0.80,0.10,0.10)(0.90,0.10,0.00\right) \end{array}\right]\\ C_{1}=\left[\begin{array}{l} \left(0.25,0.60,0.15)(0.10,0.90,0.00)(0.50,0.40,0.10)(1.00,0.00,0.00)(0.25,0.60,0.15)(0.40,0.55,0.05\right)\\ \left(0.80,0.10,0.10)(0.80,0.10,0.10)(0.90,0.10,0.00)(0.90,0.10,0.00)(0.80,0.10,0.10)(0.70,0.20,0.10\right)\\ \left(0.70,0.20,0.10)(0.60,0.30,0.10)(0.60,0.30,0.10)(0.80,0.10,0.10)(0.70,0.20,0.10)(0.80,0.10,0.10\right) \end{array}\right]\\ D_{1}=\left[\begin{array}{l} \left(0.80,0.10,0.10)(0.90,0.10,0.00)(0.80,0.10,0.10)(0.10,0.90,0.00)(0.10,0.90,0.00)(0.10,0.75,0.15\right)\\ \left(0.25,0.60,0.15)(0.10,0.75,0.15)(0.10,0.75,0.15)(1.00,0.00,0.00)(0.90,0.10,0.00)(0.80,0.10,0.10\right)\\ \left(0.70,0.20,0.10)(0.50,0.40,0.10)(0.80,0.10,0.10)(0.50,0.40,0.10)(0.40,0.55,0.05)(0.70,0.20,0.10\right) \end{array}\right]\\ E_{1}=\left[\begin{array}{l} \left(0.80,0.10,0.10)(0.60,0.30,0.10\right)\\ \left(0.70,0.20,0.10)(1.00,0.00,0.00\right)\\ \left(0.90,0.10,0.00)(1.00,0.00,0.00\right) \end{array}\right] \end{array}$$

$$\begin{array}{l} R_{2}=[A_{2}B_{2}C_{2}D_{2}E_{2}]\\ A_{2}=\left[\begin{array}{l} \left(0.80,0.10,0.10)(0.90,0.10,0.00)(0.60,0.30,0.10)(0.80,0.10,0.10)(0.70,0.20,0.10)(0.50,0.40,0.10\right)\\ \left(0.80,0.10,0.10)(0.90,0.10,0.00)(0.80,0.10,0.10)(0.90,0.10,0.00)(0.60,0.30,0.10)(0.80,0.10,0.10\right)\\ \left(0.10,0.75,0.15)(0.40,0.55,0.05)(0.90,0.10,0.00)(0.80,0.10,0.10)(0.60,0.30,0.10)(0.80,0.10,0.10\right) \end{array}\right]\\ B_{2}=\left[\begin{array}{l} \left(0.80,0.10,0.10)(0.80,0.10,0.10)(0.80,0.10,0.10)(0.80,0.10,0.10)(0.70,0.20,0.10)(0.10,0.75,0.15\right)\\ \left(0.80,0.10,0.10)(0.70,0.20,0.10)(0.90,0.10,0.00)(0.60,0.30,0.10)(0.50,0.40,0.10)(0.80,0.10,0.10\right)\\ \left(0.80,0.10,0.10)(0.80,0.10,0.10)(0.80,0.10,0.10)(0.80,0.10,0.10)(0.80,0.10,0.10)(0.80,0.10,0.10\right) \end{array}\right]\\ C_{2}=\left[\begin{array}{l} \left(0.25,0.60,0.15)(0.10,0.75,0.15)(0.50,0.40,0.10)(1.00,0.00,0.00)(0.25,0.60,0.15)(0.40,0.55,0.05\right)\\ \left(0.90,0.10,0.00)(0.80,0.10,0.10)(0.90,0.10,0.00)(0.90,0.10,0.00)(0.80,0.10,0.10)(0.70,0.20,0.10\right)\\ \left(0.70,0.20,0.10)(0.60,0.30,0.10)(0.60,0.30,0.10)(0.80,0.10,0.10)(0.70,0.20,0.10)(0.80,0.10,0.10\right) \end{array}\right]\\ D_{2}=\left[\begin{array}{l} \left(0.80,0.10,0.10)(0.80,0.10,0.10)(0.90,0.10,0.00)(0.10,0.90,0.00)(0.10,0.75,0.15)(0.10,0.75,0.15\right)\\ \left(0.40,0.55,0.05)(0.10,0.75,0.15)(0.10,0.90,0.00)(1.00,0.00,0.00)(0.90,0.10,0.00)(0.80,0.10,0.10\right)\\ \left(0.70,0.20,0.10)(0.80,0.10,0.10)(0.70,0.20,0.10)(0.50,0.40,0.10)(0.40,0.55,0.05)(0.70,0.20,0.10\right) \end{array}\right]\\ E_{2}=\left[\begin{array}{l} \left(0.80,0.10,0.10)(0.50,0.40,0.10\right)\\ \left(0.70,0.20,0.10)(0.90,0.10,0.00\right)\\ \left(0.90,0.10,0.00)(0.90,0.10,0.00\right) \end{array}\right] \end{array}$$

R3=[A3B3C3D3E]3A3=[(0.80,0.10,0.10)(0.90,0.10,0.00)(0.70,0.20,0.10)(0.80,0.10,0.10)(0.70,0.20,0.10)(0.60,0.30,0.10)(0.80,0.10,0.10)(0.90,0.10,0.00)(0.70,0.20,0.10)(0.90,0.10,0.00)(0.60,0.30,0.10)(0.80,0.10,0.10)(0.10,0.75,0.15)(0.10,0.75,0.15)(0.90,0.10,0.00)(0.80,0.10,0.10)(0.60,0.30,0.10)(0.90,0.10,0.00)]B3=[(0.80,0.10,0.10)(0.80,0.10,0.10)(0.90,0.10,0.00)(0.80,0.10,0.10)(0.70,0.20,0.10)(0.10,0.75,0.15)(0.80,0.10,0.10)(0.60,0.30,0.10)(0.90,0.10,0.00)(0.60,0.30,0.10)(0.50,0.40,0.10)(0.80,0.10,0.10)(0.90,0.10,0.00)(0.70,0.20,0.10)(0.70,0.20,0.10)(0.80,0.10,0.10)(0.80,0.10,0.10)(0.90,0.10,0.00)]C3=[(0.40,0.55,0.05)(0.10,0.75,0.15)(0.50,0.40,0.10)(1.00,0.00,0.00)(0.40,0.55,0.05)(0.40,0.55,0.05)(0.90,0.10,0.00)(0.80,0.10,0.10)(0.90,0.10,0.00)(0.90,0.10,0.00)(0.80,0.10,0.10)(0.50,0.40,0.10)(0.70,0.20,0.10)(0.60,0.30,0.10)(0.60,0.30,0.10)(0.80,0.10,0.10)(0.70,0.20,0.10)(0.80,0.10,0.10)]D3=[(0.80,0.10,0.10)(0.80,0.10,0.10)(0.90,0.10,0.00)(0.10,0.90,0.00)(0.10,0.75,0.15)(0.10,0.75,0.15)(0.10,0.75,0.15)(0.10,0.75,0.15)(0.10,0.90,0.00)(1.00,0.00,0.00)(0.90,0.10,0.00)(0.80,0.10,0.10)(0.70,0.20,0.10)(0.80,0.10,0.10)(0.90,0.10,0.00)(0.80,0.10,0.10)(0.25,0.60,0.15)(0.70,0.20,0.10)]E3=[(0.90,0.10,0.00)(0.60,0.30,0.10)(0.80,0.10,0.10)(1.00,0.00,0.00)(0.90,0.10,0.00)(0.90,0.10,0.00)]

 (2) The weight of the DMs is determined using the entropy method.

According to Equations (10)–(12), the weight of the DMs can be obtained:

$$E^{IFS}(R_{1})=\frac{1}{3\times 26\times \ln2}\sum_{i=1}^{3}\sum_{j=1}^{26}\left[\mu_{ij}\ln\mu_{ij}+\nu_{ij}\ln\nu_{ij}-(1-\pi_{ij})\ln(1-\pi_{ij})-\pi_{ij}\ln2\right]$$

$$w_{d}^{1}=\frac{d_{R_{1}}}{\sum_{k=1}^{3}d_{R_{k}}}=0.334$$

By the same token,

$$w_{d}^{2}=\frac{d_{R_{2}}}{\sum_{k=1}^{3}d_{R_{k}}}=0.323$$

$$w_{d}^{3}=\frac{d_{R_{3}}}{\sum_{k=1}^{3}d_{R_{k}}}=0.343$$

By calculating the above equation, the set of decision-maker weights can be obtained:

$$w_{d}=\left\{0.334,0.323,0.343\right\}$$

2. An intuitionistic fuzzy decision matrix is established to determine the weight of the criteria.

According to the calculation of Equations (13)–(17), the combined weights of the criteria are calculated according to Equation (18), as shown in Table 7.

3. Using IFHPWA, the comprehensive evaluation of the alternatives is integrated.

Because the 7 primary and 26 secondary criteria are all benefit criteria, there is no need to normalize the individual decision-making matrix.

The comprehensive evaluation decision matrix is obtained by Equations (19)–(21), as shown in Table 8.

4. The alternatives are ranked using the TOPSIS method.

The intuitionistic fuzzy positive ideal solution $Z_{j}^{+}$ and the intuitionistic fuzzy negative ideal solution $Z_{j}^{-}$ can be obtained through Equations (22) and (23), as shown in Table 9.

The Euclidean distance between the alternative suppliers Z_1_, Z_2_, and Z_3_ and the positive and negative ideal solutions are calculated by Equations (24) and (25):

$$Z_{1}:\;d_{1}^{+}=0.626;\;d_{1}^{-}=0.746$$

$$Z_{2}:\;d_{2}^{+}=0.349;\;d_{2}^{-}=0.602$$

$$Z_{3}:\;d_{3}^{+}=0.351;\;d_{3}^{-}=0.522$$

The relative closeness coefficient of alternative suppliers can then be computed by Equation (26) and the best supplier selected:


Z_1_: CC_1_ = 0.544

Z_2_: CC_2_ = 0.633

Z_3_: CC_3_ = 0.598

From the above calculation and ranking, alternative supplier Z_2_ is the optimal supplier, and Enterprise A should choose supplier Z_2_ as its emergency epidemic material supplier.

### 6.3. Comparison

**Figure 3:**
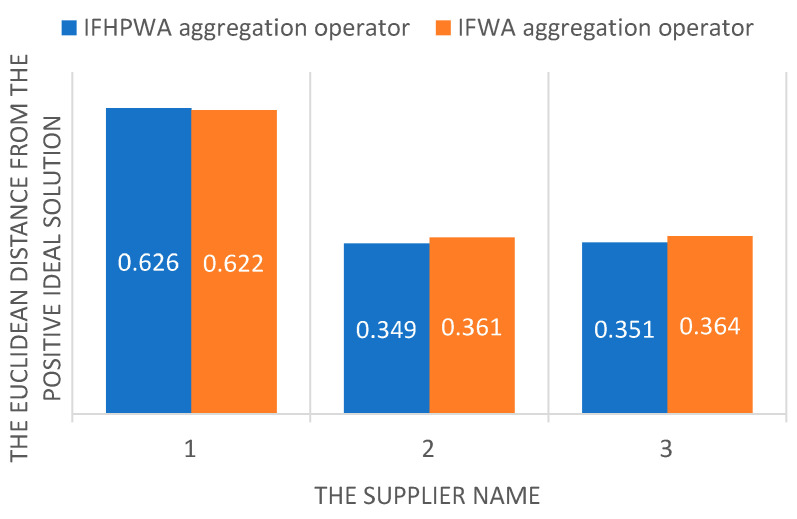
Euclidean distance between the alternative suppliers and the positive ideal solution.

**Figure 4:**
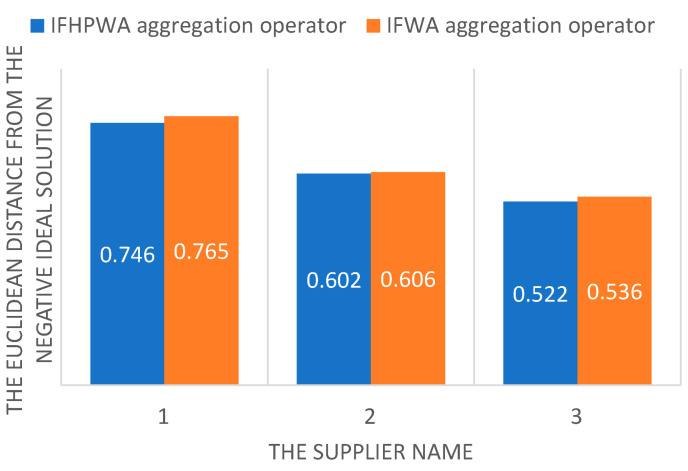
Euclidean distance between the alternative suppliers and the negative ideal solution.

We compared the aggregation effect of the IFHPWA operator and IFWA operator for decision information in emergency decision making to verify the feasibility and application value of this study. The corresponding calculation and analysis were based on the same emergency decision scenario mentioned above. For the weighted decision information, we used the IFHPWA operator and IFWA operator for aggregation and then used the TOPSIS method to rank the aggregated comprehensive decision information. As shown in Figure 3 and Figure 4, adding consideration of the decision makers’ decision sequence will shorten the distance between the alternative evaluation and the positive and negative ideal solutions, which validates the proposed conjecture that the decision result of the upper decision maker will affect the decision result of the lower decision maker to a certain extent in a real decision environment. Therefore, the addition of the IFHPWA operator will bring the decision-making process closer to the reality of emergency decision making.

**Figure 5:**
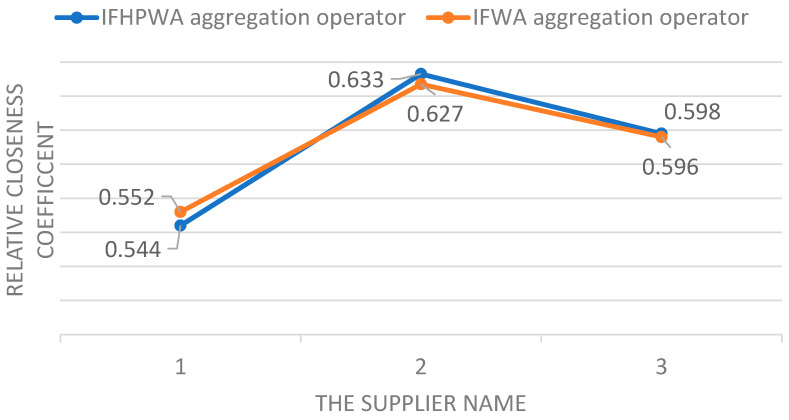
Relative closeness coefficient of the alternatives.

The alternatives can be ranked according to the relative closeness coefficient calculated by the TOPSIS method. Based on Figure 5, adding the IFHPWA operator can effectively expand the relative closeness coefficient’s distance between alternatives, help reduce the hesitation of decision makers regarding alternatives, improve decision-making efficiency and feasibility, and meet the characteristic requirements of emergency decision making.

## 7. Discussion

### 7.1. Theoretical Implications

Based on the background of the public health emergency caused by COVID-19, this paper studies the selection of emergency material suppliers by using the MCGDM method. This research extends the MCGDM method in the following ways: (1) We put forward a new decision-making framework. The framework considers the current demand of the emergency decision environment, which simplifies the decision steps and ensures accurate and scientific selection, which can effectively solve realistic emergency issues. (2) We proposed a novel aggregation operator. The IFHPWA operator can effectively expand the relative closeness coefficient’s distance between alternatives and help reduce the hesitation degree of decision makers regarding alternatives; therefore, application of the IFHPWA operator improves the efficiency and feasibility of decision making to meet the characteristic requirements of emergency decision making. (3) We proposed an overall and widely applicable emergency material supplier evaluation criteria system, which includes seven primary criteria and twenty-six secondary criteria. Thus, the evaluation results can better fit a realistic emergency issue.

### 7.2. Practical Implications

The MCGDM method is widely used in the study of emergency decision making and has been proved to be a decision-making method with both theoretical and practical value. This study puts forward several implications for emergency decision making: (1) Fully considering the real environment of emergency decision-making. Emergency decision making needs to be fast and accurate [14]. Compared to some emergency decision-making methods [14,16], the decision-making framework proposed here considers the actual impact of the decision order of decision makers on the decision results, and the decision-making steps are relatively short; thus, it fully meets the needs of emergency decision making. (2) Evaluation criteria and their weight should be made to be more reasonable. In order to better fit the decision-making problem and consider actual decision-making needs, we put forward a relatively comprehensive evaluation criteria system, used the AHP method to weight the two-level evaluation criteria, and finally solved the combined criteria weights. Compared to the DEMATEL method and BWM method [58,60], this method better passes the importance of the primary criteria to the secondary criteria, which makes the weight of evaluation criteria more applicable to emergency decision making. (3) This method can be extended to other decision-making methods or decision-making environments. Although the MCGDM method proposed in this paper is more suitable for emergency decision environments, it is convenient for the expansion of research and method reconstruction in the future because of its distinctive sub-method characteristics and simple combination.

### 7.3. Limitation and Future Work

The method proposed in this paper still has some limitations. (1) The weighting method for criteria is traditional, belonging to the subjective weight method, which is easily influenced by the personal preferences of decision makers. (2) The IFHPWA operator cannot independently rank alternatives and still needs to be combined with a ranking method. In future research, we will carry out our work from the following three aspects: (1) we will improve the reliability of criteria weighting by combining subjective and objective weighting methods; (2) we will improve the IFHPWA operator and make it rank all alternatives independently, thereby simplifying the decision-making process and improving decision-making efficiency; and (3) we will attempt to introduce a loss function to optimize the decision-making priority coefficient calculation process, simplify its calculation steps, and improve its accuracy in describing decision makers’ priorities.

## 8. Conclusions

Public health emergencies such as COVID-19 have produced many social concerns. Public health emergencies are sudden and complex, and they can be accompanied by a sharp increase in the demand for emergency materials. In response to such events, enterprises must choose emergency medical material suppliers as soon as possible to ensure normal production.

In this case, we proposed an extended MCGDM method and corresponding evaluation framework for emergency medical material suppliers. We proposed an evaluation system that includes 7 primary and 26 secondary evaluation criteria, which are evaluated by intuitionistic fuzzy sets. We then used the AHP method and entropy method to weight the evaluation criteria and decision makers. Finally, we proposed the IFHPWA operator, which considers the decision sequence of decision makers to aggregate decision information, and used the TOPSIS method to rank the suppliers.

## Figures and Tables

**Table 1:** Representative methods in emergency decision studies.

References	Research Field	Method
Zhang et al. [18]	Public Emergency	MCGDM
Qin et al. [14]	Public Health Emergency	MCGDM
Liu et al. [16]	Rescue in Earthquake-Stricken Areas	Dynamical Consensus Method
Yang et al. [37]	Sudden Accidents	Bayesian Inference
Zheng et al. [42]	Typhoon Disaster Assessment	MCGDM
Ding et al. [43]	Public Health Emergency	MCGDM
Nassereddine et al. [41]	Emergency Response	MCGDM

**Table 2:** Evaluation criteria system for emergency material suppliers.

Primary Criteria	Secondary Criteria	Primary Criteria	Secondary Criteria
Enterprise credit	financial status	Supply capacity	order completion rate
	development prospects		packing sound rate
	cooperation attitude		delivery punctuality
	industry status and reputation	Flexibility	batch flexibility
	employee quality		category flexibility
	equipment level		time flexibility
	risk management ability	Price	price stability
	informatization degree		price advantage
Rapid response ability	information exchange capacity	Enterprise environment	economic environment
	emergency distribution capacity		natural environment
	order processing ability		political environment
Quality	return processing capacity		
	qualified rate		
	quality certification		
	total quality management ability		

**Table 3:** Symbols/collections in this document.

Notations	Definitions
$A=\left\{A_{1},A_{2},\cdots ,A_{m}\right\},1\leq i\leq m$	Set of alternative suppliers
$C=\left\{C_{1},C_{2},\cdots ,C_{n}\right\},1\leq j\leq n,1\leq h\leq n$	Set of criteria
$\alpha =\left\{\alpha_{1},\alpha_{2},\cdots ,\alpha_{p}\right\},1\leq k\leq p$	Set of DMs
$R_{k}$	Individual decision matrix
$R^{k}$	Individual decision matrix of criteria weighting
$R_{d}^{k}$	The weighted individual decision matrix
R	Group intuitionistic fuzzy decision matrix
$E^{IFS}(R^{k})$	Entropy value of $\alpha_{k}$
$d_{R^{k}}$	Degree of divergence
$w_{d}^{k}$	Weight of $\alpha_{k}$
$w_{d}=\left\{w_{d}^{1},w_{d}^{2},\cdots ,w_{d}^{k}\right\}$	Set of the weights of decision makers
$\bar{R}$	Consistency test value of subjective weight
ξ	Threshold for the consistency test, generally taken as ξ=1
$w_{jh}$	Combined weight of the fuzzy-AHP method
$w_{c_{j}}$	Weight of primary criteria in the fuzzy-AHP method
$w_{c_{jh}}$	Weight of secondary criteria in the fuzzy-AHP method
D	Comprehensive evaluation matrix
$Z_{j}^{+}$	Intuitionistic fuzzy positive ideal solution
$Z_{j}^{-}$	Intuitionistic fuzzy negative ideal solution
$d_{i}^{+}(i=1,2,\cdots ,m)$	Distance between $A_{i}$ and the positive ideal solution
$d_{i}^{-}(i=1,2,\cdots ,m)$	Distance between $A_{i}$ and the negative ideal solution
$CC_{i}(i=1,2,\cdots ,m)$	Relative closeness coefficient of $A_{i}$

**Table 4:** Procurement list of emergency anti-epidemic goods.

Definition	Specification	Number
Infrared Thermometer	General	500
Goggles	Medical	8000
Mask	Medical	30,000
Full Protective Clothing	Medical	25,000
Disinfectant	25 kg	500
75% Medical Alcohol	1500 mL	5000
Disposable Gloves	Medical	30,000
Hand Gel	500 mL	50,000
Disinfection Spray	Back-Carrying Type	500

**Table 5:** Set of language variables used for DM evaluations.

Language Variables	Corresponding Intuitionistic Fuzzy Number
Excellent (E)	(1.00, 0.00, 0.00)
Very, Very Good (VVG)	(0.90, 0.10, 0.00)
Very Good (VG)	(0.80, 0.10, 0.10)
Good (G)	(0.70, 0.20, 0.10)
Medium Good (MG)	(0.60, 0.30, 0.10)
Medium (M)	(0.50, 0.40, 0.10)
Medium Bad (MB)	(0.40, 0.55, 0.05)
Bad (B)	(0.25, 0.60, 0.15)
Very Bad (VB)	(0.10, 0.75, 0.15)
Very, Very Bad (VVB)	(0.10, 0.90, 0.00)

**Table 6:** Individual evaluation results.

Criteria	$\alpha_{1}$			$\alpha_{2}$			$\alpha_{3}$		
	Z_1_	Z_2_	Z_3_	Z_1_	Z_2_	Z_3_	Z_1_	Z_2_	Z_3_
C_11_	VG	VG	B	VG	VG	VB	VG	VG	VB
C_12_	VG	VG	M	VVG	VVG	MB	VVG	VVG	VB
C_13_	G	VG	VVG	MG	VG	VVG	G	G	VVG
C_14_	VG	VVG	VG	VG	VVG	VG	VG	VVG	VG
C_15_	G	M	G	G	MG	MG	G	MG	MG
C_16_	M	VG	VG	M	VG	VG	MG	VG	VVG
C_17_	VG	VG	G	VG	VG	VG	VG	VG	VVG
C_18_	VVG	G	VG	VG	G	VG	VG	MG	G
C_21_	VG	VVG	VG	VG	VVG	VG	VVG	VVG	G
C_22_	VVG	G	VG	VG	MG	VG	VG	MG	VG
C_23_	G	M	VG	G	M	VG	G	M	VG
C_31_	VVB	VG	VVG	VB	VG	VG	VB	VG	VVG
C_32_	B	VG	G	B	VVG	G	MB	VVG	G
C_33_	VVB	VG	MG	VB	VG	MG	VB	VG	MG
C_34_	M	VVG	MG	M	VVG	MG	M	VVG	MG
C_41_	E	VVG	VG	E	VVG	VG	EG	VVG	VG
C_42_	B	VG	G	B	VG	G	MB	VG	G
C_43_	MB	G	VG	MB	G	VG	MB	M	VG
C_51_	VG	B	G	VG	MB	G	VG	VB	G
C_52_	VVG	VB	M	VG	VB	VG	VG	VB	VG
C_53_	VG	VB	VG	VVG	VVB	G	VVG	VVB	VVG
C_61_	VVB	E	M	VVB	E	M	VVB	E	VG
C_62_	VVB	VVG	MB	VB	VVG	MB	VB	VVG	B
C_71_	VB	VG	G	VB	VG	G	VB	VG	G
C_72_	VG	G	VVG	VG	G	VVG	VVG	VG	VVG
C_73_	MG	E	E	M	VVG	VVG	MG	E	VVG

**Table 7:** Combined weights of the criteria.

	Combined Weights				Combined Weights		
$W_{11}$	0.043	0.940	0.017	$W_{33}$	0.033	0.952	0.015
$W_{12}$	0.029	0.957	0.014	$W_{34}$	0.033	0.952	0.015
$W_{13}$	0.035	0.950	0.015	$W_{41}$	0.030	0.956	0.015
$W_{14}$	0.024	0.961	0.015	$W_{42}$	0.029	0.957	0.015
$W_{15}$	0.034	0.951	0.015	$W_{43}$	0.030	0.955	0.015
$W_{16}$	0.030	0.964	0.006	$W_{51}$	0.028	0.957	0.014
$W_{17}$	0.039	0.945	0.016	$W_{52}$	0.027	0.958	0.014
$W_{18}$	0.031	0.954	0.015	$W_{53}$	0.030	0.956	0.014
$W_{21}$	0.029	0.957	0.014	$W_{61}$	0.030	0.955	0.015
$W_{22}$	0.033	0.951	0.015	$W_{62}$	0.023	0.963	0.014
$W_{23}$	0.029	0.957	0.015	$W_{71}$	0.038	0.946	0.016
$W_{31}$	0.030	0.955	0.015	$W_{72}$	0.032	0.953	0.015
$W_{32}$	0.035	0.949	0.015	$W_{73}$	0.038	0.946	0.016

**Table 8:** Comprehensive evaluation decision matrix.

	**C_11_**			**C_12_**			**C_13_**			**C_14_**		
Z_1_	0.230	0.655	0.115	0.175	0.729	0.096	0.162	0.751	0.087	0.145	0.749	0.106
Z_2_	0.230	0.655	0.115	0.175	0.729	0.095	0.196	0.699	0.105	0.166	0.749	0.084
Z_3_	0.057	0.883	0.059	0.094	0.848	0.058	0.229	0.697	0.074	0.145	0.749	0.106
	**C_15_**			**C_16_**			**C_17_**			**C_18_**		
Z_1_	0.163	0.747	0.089	0.101	0.858	0.041	0.214	0.675	0.111	0.201	0.715	0.084
Z_2_	0.114	0.820	0.065	0.174	0.765	0.061	0.214	0.675	0.111	0.151	0.759	0.089
Z_3_	0.158	0.755	0.087	0.175	0.765	0.060	0.190	0.715	0.094	0.178	0.717	0.105
	**C_21_**			**C_22_**			**C_23_**			**C_31_**		
Z_1_	0.170	0.729	0.100	0.211	0.701	0.087	0.144	0.770	0.086	0.019	0.976	0.005
Z_2_	0.195	0.730	0.074	0.155	0.755	0.090	0.098	0.840	0.062	0.174	0.720	0.106
Z_3_	0.167	0.733	0.100	0.188	0.701	0.111	0.169	0.729	0.101	0.197	0.720	0.083
	**C_32_**			**C_33_**			**C_34_**			**C_41_**		
Z_1_	0.053	0.889	0.058	0.020	0.975	0.005	0.108	0.828	0.064	0.231	0.678	0.091
Z_2_	0.210	0.693	0.096	0.188	0.706	0.106	0.219	0.706	0.076	0.202	0.725	0.074
Z_3_	0.166	0.741	0.092	0.133	0.791	0.075	0.133	0.791	0.075	0.174	0.725	0.101
	**C_42_**			**C_43_**			**C_51_**			**C_52_**		
Z_1_	0.047	0.899	0.054	0.079	0.882	0.039	0.053	0.889	0.058	0.020	0.975	0.005
Z_2_	0.169	0.729	0.101	0.147	0.764	0.089	0.210	0.693	0.096	0.188	0.706	0.106
Z_3_	0.144	0.770	0.086	0.174	0.720	0.106	0.166	0.741	0.092	0.133	0.791	0.075
	**C_53_**			**C_61_**			**C_62_**					
Z_1_	0.108	0.828	0.064	0.231	0.678	0.091	0.047	0.899	0.054			
Z_2_	0.219	0.706	0.076	0.202	0.725	0.074	0.169	0.729	0.101			
Z_3_	0.133	0.791	0.075	0.174	0.725	0.101	0.144	0.770	0.086			
	**C_71_**			**C_72_**			**C_73_**					
Z_1_	0.079	0.882	0.039	0.185	0.710	0.105	0.143	0.781	0.076			
Z_2_	0.147	0.764	0.089	0.157	0.753	0.090	0.276	0.632	0.092			
Z_3_	0.174	0.720	0.106	0.212	0.710	0.078	0.273	0.636	0.091			

**Table 9:** Positive and negative ideal solution values.

Criteria	$Z_{j}^{+}$	$Z_{j}^{-}$
C_11_	(0.230, 0.655, 0.115)	(0.057, 0.883, 0.059)
C_12_	(0.175, 0.729, 0.095)	(0.094, 0.848, 0.058)
C_13_	(0.229, 0.697, 0.074)	(0.162, 0.751, 0.087)
C_14_	(0.166, 0.749, 0.084)	(0.145, 0.749, 0.106)
C_15_	(0.163, 0.747, 0.089)	(0.114, 0.820, 0.065)
C_16_	(0.175, 0.765, 0.060)	(0.101, 0.858, 0.041)
C_17_	(0.214, 0.675, 0.111)	(0.190, 0.715, 0.094)
C_18_	(0.201, 0.715, 0.084)	(0.151, 0.759, 0.089)
C_21_	(0.195, 0.730, 0.074)	(0.167, 0.733, 0.100)
C_22_	(0.211, 0.701, 0.087)	(0.155, 0.755, 0.090)
C_23_	(0.169, 0.729, 0.101)	(0.144, 0.770, 0.086)
C_31_	(0.197, 0.720, 0.083)	(0.019, 0.976, 0.005)
C_32_	(0.210, 0.693, 0.096)	(0.053, 0.889, 0.058)
C_33_	(0.188, 0.706, 0.106)	(0.020, 0.975, 0.005)
C_34_	(0.219, 0.706, 0.076)	(0.108, 0.828, 0.064)
C_41_	(0.231, 0.678, 0.091)	(0.174, 0.725, 0.101)
C_42_	(0.169, 0.729, 0.101)	(0.047, 0.899, 0.054)
C_43_	(0.174, 0.720, 0.106)	(0.079, 0.882, 0.039)
C_51_	(0.165, 0.729, 0.106)	(0.047, 0.889, 0.054)
C_52_	(0.179, 0.734, 0.086)	(0.0 17, 0.940, 0.042)
C_53_	(0.180, 0.725, 0.095)	(0.019, 0.940, 0.041)
C_61_	(0.231, 0.672, 0.097)	(0.019, 0.976, 0.005)
C_62_	(0.160, 0.760, 0.080)	(0.015, 0.979, 0.006)
C_71_	(0.210, 0.679, 0.111)	(0.022, 0.932, 0.047)
C_72_	(0.212, 0.710, 0.078)	(0.157, 0.753, 0.090)
C_73_	(0.276, 0.632, 0.092)	(0.143, 0.781, 0.076)

## Data Availability

The data used to support the findings of this study are included within the article.
